# Acclimation Time Enhances Adaptation of Heterotrophic Nitrifying-Aerobic Denitrifying Microflora to Linear Anionic Surfactant Stress

**DOI:** 10.3390/microorganisms13051031

**Published:** 2025-04-29

**Authors:** Huihui Han, Peizhen Chen, Wenjie Zhao, Shaopeng Li, Keyu Zhang

**Affiliations:** 1 Agro-Environmental Protection Institute, Ministry of Agriculture and Rural Affairs, Tianjin 300191, China; mymymingyou@163.com (H.H.); zwj1997jc@163.com (W.Z.);; 2 College of Resources and Environment, Huazhong Agricultural University, Wuhan 430070, China; 3 College of Agriculture & Resources and Environment, Tianjin Agricultural University, Tianjin 300392, China; lishaopeng518896@163.com

**Keywords:** heterotrophic nitrification-aerobic denitrification (HN-AD), microbial adaptation, linear anionic surfactant (LAS), metagenomic analysis, stress response, nitrogen removal

## Abstract

Linear anionic surfactants (LAS) pose significant stress to microbial denitrification in wastewater treatment. This study investigated the performance and adaptation mechanisms of heterotrophic nitrification-aerobic denitrification (HN-AD) microbial consortia under LAS exposure after short-term (SCM, 2 months) and long-term (LCM, 6 months) acclimation. Results showed a dose-dependent inhibition of total nitrogen (TN) removal, with LCM achieving 97.40% TN removal under 300 mg/L LAS, which was 16.89% higher than SCM. Biochemical assays indicated that LCM exhibited lower reactive oxygen species (ROS) levels, a higher ATP content, and reduced LDH release, suggesting enhanced oxidative stress resistance and membrane stability. EPS secretion also increased in LCM, contributing to environmental tolerance. Metagenomic analysis revealed that long-term acclimation enriched key genera including *Pseudomonas*, *Aeromonas*, and *Stutzerimonas*, which maintained higher expression of denitrification (e.g., *nosZ*, *nirS*) and ammonium assimilation genes (*glnA*, *gltB*). Although high LAS concentrations reduced overall community diversity and led to convergence between SCM and LCM structures, LCM retained greater functional capacity and stress resistance. These findings underscore the importance of acclimation in sustaining denitrification performance under surfactant pressure and offer valuable insights for engineering robust microbial consortia in complex wastewater environments.

## 1. Introduction

Rapid urbanization and industrialization have led to the discharge of large quantities of domestic and industrial wastewater into natural water bodies, resulting in excessive accumulation of nitrogen-containing compounds in aquatic ecosystems. This accumulation triggers eutrophication, disrupts ecological balance, and adversely affects human health [1]. While physicochemical methods have been somewhat effective in nitrogen pollution treatment, their high costs and potential for secondary pollution make biological nitrogen removal methods a more attractive alternative [2]. In the traditional biological nitrogen removal process, the nitrification and denitrification processes are performed by autotrophic nitrifying bacteria and heterotrophic denitrifying bacteria under different environmental conditions [3]. Due to the different environmental requirements of these microorganisms, conventional processes typically necessitate separate nitrification and denitrification stages, complicating infrastructure and prolonging treatment times [4]. Heterotrophic nitrification-aerobic denitrification (HN-AD) bacteria provide a promising solution, as they can simultaneously perform nitrification and denitrification under aerobic conditions, simplifying the treatment process and reducing operational costs [5]. Therefore, HN-AD bacteria have become a focal point in biological nitrogen removal research [6]. However, maintaining the stability and high efficiency of these HN-AD bacteria under complex environmental conditions in practical applications remains challenging.

Linear anionic surfactants (LAS), a major category of anionic surfactants, are widely used in household detergents and cleaners, and they are frequently found in wastewater due to their high usage [7]. LAS concentrations exceeding 0.5 mg/L can cause persistent foaming on water surfaces, forming a barrier that impedes oxygen transfer between water and air, thus lowering dissolved oxygen levels and degrading water quality [8]. The concentration of LAS in untreated domestic sewage has been reported to range from 1 to 18 mg/L, while in commercial laundry wastewater, LAS concentrations can exceed 300 mg/L [9,10]. When high levels of LAS are introduced into wastewater treatment plants, they may disrupt biological treatment processes. Studies indicate that LAS concentrations above 10 mg/L inhibit the denitrification efficiency of aerobic denitrifying bacteria by reducing nitrate reductase (NR) and nitrite reductase (NiR) activities [11,12]. Additionally, LAS negatively impacts the metabolic activity and cell membrane integrity of anaerobic ammonium-oxidizing bacteria. Furthermore, LAS at concentrations of 15 mg/L has been shown to significantly affect microbial diversity in denitrifying bioreactors [13]. These findings suggest that LAS impairs the physiological activities of nitrogen-removing microorganisms, hindering their nitrogen removal capacity [14].

In recent years, researchers have begun to focus on optimizing the composition and function of indigenous complex microflora including HN-AD consortia, to improve their adaptability and stability under complex environmental conditions [15]. Domestication can gradually adapt the microflora to specific environmental conditions through selective pressure and eventually form microbial communities with higher functional stability and environmental adaptability [16]. These composite microflora have demonstrated significant advantages in multiple applications such as pollutant degradation, resource recovery, and bioremediation [17,18,19]. Studies indicate that domestication promotes the expression of essential functional genes, enabling microbial communities to exhibit greater tolerance and adaptability to environmental fluctuations [20]. Domesticated sludge-derived microbial consortia used in wastewater treatment have exhibited stable nitrogen removal performance under various stressors, including high pollutant concentrations, extreme temperatures, and pH fluctuations [21,22].

Selective domestication has been proposed to mitigate the negative effects of LAS on nitrogen-removing microorganisms, effectively enhancing the adaptability and stability of microbiota under complex environmental conditions [23]. Through domestication, microbial communities can gradually adapt to specific environmental conditions, forming consortia with high functional stability and environmental resilience. However, the domestication of HN-AD complex microflora under LAS stress remains insufficiently studied, particularly regarding the influence of acclimation duration on the resilience and stability of these communities.

Previous studies suggest that an appropriate acclimation period can significantly enhance microbial tolerance in challenging environments, such as high salinity or low temperatures, while maintaining efficient nitrogen removal performance [24]. However, under continuous exposure to LAS, the long-term adaptation mechanisms and evolutionary trajectories of entire microbial consortia remain insufficiently explored. Most existing studies have focused on individual strains or short-term stress responses, overlooking the stepwise selection of microbial community structures, the coordinated adjustment of metabolic functions, and the restructuring of ecological interaction networks under dynamic environmental pressures [25]. Therefore, investigating the adaptive evolution of complex microbial communities under sustained pollutant stress, and identifying key tolerance-related functional genes and ecological core taxa, is of great significance for constructing robust microbial agents and ensuring stable system performance in contaminated scenarios.

In this study, a heterotrophic nitrification-aerobic denitrification (HN-AD) microbial consortium was used as the model system. Two acclimation periods were set (SCM: 2 months; LCM: 6 months), along with a gradient of LAS concentrations. The study systematically evaluated the functional evolution and community restructuring of microbial consortia under long-term LAS stress, using denitrification performance, physiological indicators, and metagenomic analysis. The main focuses included: (1) the influence of acclimation time on microbial resistance to LAS; (2) the coordinated variation of key metabolic and stress-response indicators; and (3) the dynamic succession of core functional genes and their microbial hosts. This study provides theoretical insights into the ecological evolution of microbial communities under pollutant stress and offers new strategies for optimizing pollution-tolerant microbial consortia.

## 2. Material and Methods

### 2.1. HN-AD Microflora Domestication Process and Culture Medium Preparation

Raw sludge samples were collected in January and May 2023 from the aeration tank of a municipal wastewater treatment plant in Tianjin, China. After collection, the sludge was transferred into 10 L polypropylene containers equipped with an aeration system (airflow rate approximately 2 L/min) and continuously aerated at room temperature to maintain aerobic conditions.

Every 48 h, 1 L of sterile heterotrophic nitrification medium (HNM) was added to each container to partially replace the original medium. This was performed to ensure nutrient supply and waste dilution, thereby promoting the enrichment and acclimation of functional heterotrophic nitrification-aerobic denitrification (HN-AD) microbial consortia. Based on acclimation duration, two groups were established: short-term acclimation (SCM) for two months and long-term acclimation (LCM) for six months.

The HNM medium was composed as follows: carbon source, disodium succinate hexahydrate (C_4_H_4_Na_2_O_4_·6H_2_O), 1.1255 g/L; nitrogen source, ammonium sulfate ((NH_4_)_2_SO_4_), 0.0943 g/L (equivalent to an initial ammonium nitrogen concentration of 20 mg/L); and 50 mL/L of Vickers salt solution as the inorganic supplement. The Vickers solution contained: K_2_HPO_4_·3H_2_O (6.5 g/L), MgSO_4_·7H_2_O (2.5 g/L), NaCl (2.5 g/L), FeSO_4_·7H_2_O (0.05 g/L), and MnSO_4_·H_2_O (0.04 g/L). All media were sterilized by autoclaving at 121 °C for 20 min before use, and the pH was adjusted to 7.0–7.2 using 1 mol/L HCl or NaOH. No additional substances were added during the acclimation process.

### 2.2. Nitrogen Removal Efficiency of HN-AD Microflora Under LAS Stress

A total of 200 mL of HNM medium (composition identical to 2.1) was added to 500 mL Erlenmeyer flasks, followed by the addition of sodium dodecylbenzenesulfonate (SDBS, chemical formula: C_18_H_29_NaO_3_S) at different concentrations to establish seven treatment groups. The final concentrations of SDBS were set at 0, 1, 5, 10, 50, 100, and 300 mg/L, labeled as LAS-0, LAS-1, LAS-5, LAS-10, LAS-50, LAS-100, and LAS-300, respectively. A 1000 mg/L SDBS stock solution was prepared using deionized water and added to the HNM during medium preparation, followed by thorough mixing to ensure uniform LAS concentration in each treatment.

Each group was prepared in triplicate. After autoclaving at 121 °C for 20 min, the flasks were inoculated in a biosafety cabinet with 20 mL of either SCM or LCM microbial suspension. The flasks were sealed with sterile, gas-permeable membranes and incubated at 37 °C on a rotary shaker at 160 rpm to maintain homogeneous dissolved oxygen levels.

Samples (8 mL each) were collected at 0, 3, 6, 9, 12, 15, 18, 24, 36, 48, and 60 h during incubation. Each sample was filtered through a 0.45 μm PTFE membrane and stored at 4 °C for subsequent nitrogen species analysis. The concentrations of NH_4_^+^-N, NO_2_^−^-N, NO_3_^−^-N, and TN were determined using a continuous flow analyzer (SEAL Analytical AA3, Ludwigshafen, Germany), following the manufacturer’s instructions.

The TN removal efficiency was calculated from Equation (1):

(1) $$Rv\left(\%\right)=\frac{T_{0}-T_{x}}{T_{x}}\times 100\%$$

where Rv is the TN removal efficiency, T_0_ is the initial TN concentration in the medium, and T_x_ is the concentration at time x hours (where x = 0, 1, 5, 10, 50, 100, 300).

### 2.3. Determination of Oxidative Stress and Cell Viability

At the end of the reaction, samples were collected from SCM and LCM cultures under LAS-0, LAS-1, LAS-10, and LAS-300 treatments. Each condition was prepared in three biological replicates. The levels of reactive oxygen species (ROS), adenosine triphosphate (ATP), and lactate dehydrogenase (LDH) were determined using commercial assay kits provided by Solarbio Life Sciences (Beijing, China). The specific product catalog numbers were as follows: ROS detection kit: CA1410; ATP detection kit: BC0960; LDH detection kit: BC0680. All assays were conducted according to the manufacturer’s protocols.

The LDH release rate was calculated from Equation (2):

(2) $$LDH\;release\;rate\left(\%\right)=\frac{{LDH}_{LAS-x}}{{LDH}_{LAS-0}}\times 100\%$$

where LDH_LAS-0_ and LDH_LAS-x_ represent LDH viability in the LAS-0 and LAS-x treatment groups, respectively (where x = 0, 1, 10, 300).

### 2.4. EPS Analysis

Samples were collected as described in Section 2.3, centrifuged at 8000 rpm for 5 min, and the supernatant was discarded to obtain the bacterial precipitate. Each sample was repeated in three groups. The precipitate was resuspended in PBS buffer, and extracellular polymeric substances (EPS) were extracted using the hot extraction method. PN and PS content were quantified using the modified Lorry method and phenol-sulfuric acid staining, respectively. Further structural analysis of EPS samples was performed using 3D-EEM fluorescence spectroscopy, as described by Zhang et al. [26].

### 2.5. Metagenome Sequencing

Samples were collected as described in Section 2.3, with no replicates for each treatment. Genomic DNA was extracted using a DNA Extraction Kit (K201, Bocai, Shanghai, China). The quality of extracted DNA was verified by 1% agarose gel electrophoresis. Sequencing was performed by the Majorbio Cloud Platform (https://cloud.majorbio.com/, accessed on 13 September 2023). Raw sequencing data were processed using Illumina-based next-generation sequencing technology (Illumina NovaSeq^TM^ X Plus sequencer, Illumina Corporation, San Diego, CA, USA). Quality control was performed using Fastp (https://github.com/OpenGene/fastp, accessed on 9 November 2023), which removed adapter sequences and filtered out reads shorter than 50 bp or with an average quality score below 20. High-quality reads were assembled into contigs using MEGAHIT v1.1.2 (https://github.com/voutcn/megahit, accessed on 11 November 2023), with a minimum contig length cutoff of 300 bp. Open reading frames (ORFs) were predicted from the assembled contigs using Prodigal v2.6.3 (https://github.com/hyattpd/Prodigal, accessed on 14 November 2023). Only predicted genes with nucleotide lengths ≥100 bp were retained and translated into amino acid sequences. The predicted protein sequences were clustered using CD-HIT (http://www.bioinformatics.org/cd-hit/, accessed on 17 November 2023) at a 90% identity and 90% coverage threshold to generate a non-redundant gene catalog, with the longest sequence in each cluster selected as the representative [27].

Non-redundant gene sequences were aligned with the NR and Kyoto Encyclopedia of Genes and Genomes (KEGG) databases using BLASTP (BLAST Version 2.2.28+, http://blast.ncbi.nlm.nih.gov/Blast.cgi, accessed on 2 December 2023) with an expectation value (e-value) of 10^−5^. Species and functional annotations were generated, and abundance calculations were performed based on these results [28].

### 2.6. Data Analysis

All experimental data were expressed as mean ± standard deviation (mean ± SD). One-way analysis of variance (ANOVA) was used to determine the significance of differences among multiple groups. When significant differences were observed (*p* < 0.05), Duncan’s multiple range test was applied to identify pairwise comparisons. The normality of the data was tested using the Shapiro–Wilk test, and homogeneity of variances was assessed using Levene’s test. If either assumption was violated, the Kruskal–Wallis H test was used for non-parametric comparisons.

Redundancy analysis (RDA) was employed to assess the relationships among environmental variables, microbial community composition, and nitrogen cycling functional genes. Additionally, a comparative heatmap based on Z-score normalization was constructed using selected stress-response indicators (ROS, ATP, LDH, EPS, PN, PS, PN/PS) under LAS-0 and LAS-300 conditions, in order to visually compare the overall physiological response patterns of SCM and LCM.

All statistical analyses were performed using SPSS 26.0. Graphs were generated with Origin 2024. Microbial diversity and functional annotation analyses were conducted via the Majorbio Cloud Platform (https://cloud.majorbio.com/, accessed on 20 February 2024), and visualizations were refined using Adobe Illustrator 2024. The nitrogen functional gene network was constructed using Gephi 0.10 (https://gephi.org/, accessed on 20 May 2024).

## 3. Results and Discussion

### 3.1. Nitrogen Removal Performance of HN-AD Microflora Under LAS Stress

**Figure 1:**
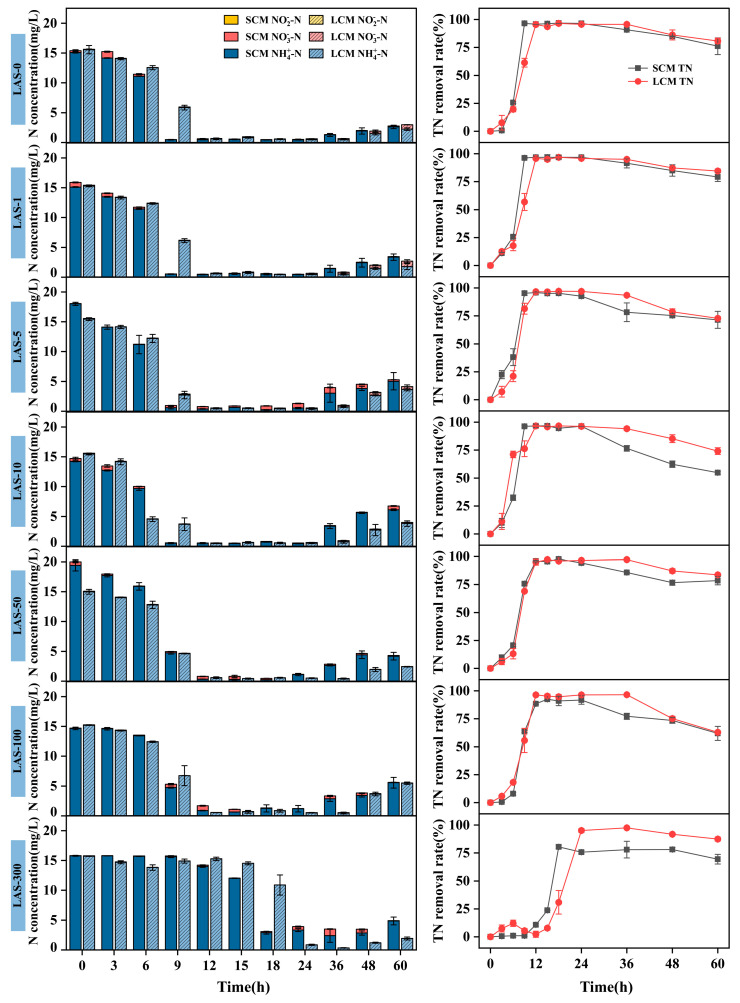
Nitrogen removal by SCM and LCM at different LAS concentrations, including NO_2_^−^-N, NO_3_^−^-N, and NH_4_^+^-N concentrations, as well as TN removal efficiency.

The nitrogen removal performance of SCM and LCM under different concentrations of LAS stress is illustrated in Figure 1. Both SCM and LCM showed an overall trend of increasing nitrogen removal rates over time, eventually reaching a stable state. In the range of 0–100 mg/L LAS concentration, both SCM and LCM could reach the maximum TN removal rate within 18 h, which were 92.42–97.61% and 96.31–97.14%, respectively, with little difference among treatment groups. However, after 36 h, the TN removal rate of LCM exceeded SCM by 3.42–19.41% higher than SCM. This finding suggests that long-term domestication enhanced the tolerance of HN-AD microflora to LAS, resolving in a greater abundance of LAS-tolerant microorganisms in LCM compared to SCM. The long-term domestication process likely improved the overall stability and functional efficiency of the microbial community by selecting and enriching stress-tolerant microorganisms [29]. At a LAS concentration of 300 mg/L, both SCM and LCM nitrogen removal capabilities were inhibited, with TN removal rates remaining nearly unchanged during the first 12 h. After 15 h, the microbial consortia gradually recovered nitrogen removal activity, with removal rates increasing rapidly. The maximum TN removal rates of SCM and LCM reached 97.40% and 80.51%, respectively. During the 24- to 60-h period, the TN removal rate of LCM was consistently 13.71–19.41% higher than SCM. This difference may be due to the formation of a high concentration LAS barrier on the liquid surface under agitation, which inhibits oxygen exchange between the HN-AD consortia and the surrounding environment [30]. Another likely factor is the toxic impact of high LAS concentrations; LAS can interact with microbial cell structures, impairing cellular functions and inhibiting microbial growth, thereby reducing nitrogen removal efficiency [12]. Over time, however, LAS-resistant or LAS-degrading HN-AD bacteria were enriched in the microflora, and LAS was increasingly degraded as a carbon source or electron acceptor, enabling the consortia to recover nitrogen removal capability [31]. Thus, following prolonged acclimation and enrichment, LCM exhibited greater stability and nitrogen removal efficiency than SCM under high LAS stress.

Throughout the experimental period, the NH_4_^+^-N removal trend was consistent with that of TN, with minimal NO_3_^−^-N accumulation and no detectable NO_2_^−^-N. This outcome can be attributed to the ability of HN-AD microflora to convert NH_4_^+^-N into N-containing gases and other substances essential for cell growth under aerobic conditions [32], further demonstrating their potential for application in the treatment of ammonia-containing wastewater. However, after 24 h, a rebound in ammonia concentration was observed over time, possibly due to the reduction of nitrate to ammonium via assimilatory or dissimilatory nitrate reduction processes under NH_4_^+^-N-limited conditions. This process provides denitrifying bacteria with the necessary substrate (ammonium), facilitating nitrogen recycling within the system [33].

### 3.2. Oxidative Stress and Cell Activity

**Figure 2:**
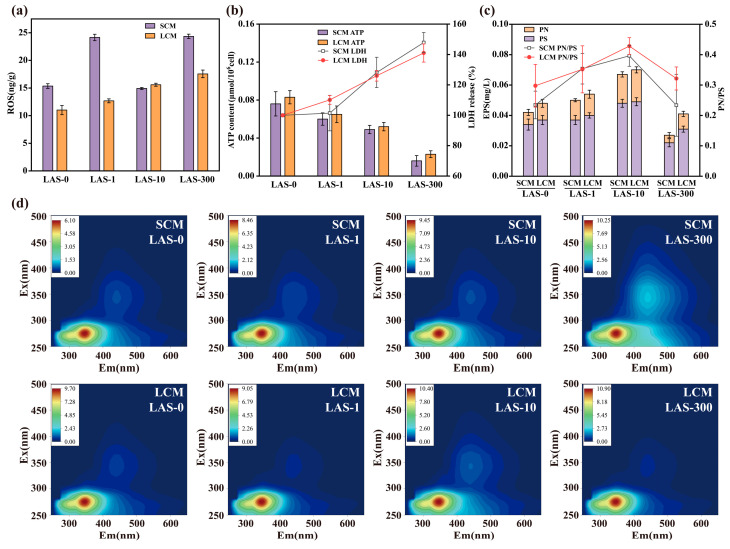
Indicators of SCM and LCM at different LAS concentrations. (**a**) ROS concentration; (**b**) ATP content and LDH release rate; (**c**) EPS content and composition; (**d**) three-dimensional fluorescence spectra.

To evaluate the biotoxic effects of LAS, we measured the levels of ROS, ATP, and LDH in samples treated with different concentrations of LAS. Notable differences were observed in ROS concentration, ATP content, and LDH release rates between microflora with different domestication durations. Reactive oxygen species (ROS) are harmful by-products of oxygen metabolism in microorganisms [34]. Under normal physiological conditions, microorganisms utilize intracellular antioxidant systems to manage lower levels of ROS [35]. However, exposure to toxic stress can lead to excessive ROS production, resulting in enzyme inactivation, protein oxidation, and DNA damage, ultimately impairing normal microbial functions [36]. The results indicated that in SCM, ROS levels increased by 57.34% and 58.59% at LAS concentrations of 1 mg/L and 300 mg/L, respectively, compared to LAS-0. Conversely, ROS levels decreased by 2.87% at a LAS concentration of 10 mg/L (Figure 2a), indicating an unstable response of SCM to varying LAS concentrations. In contrast, LCM exhibited a steady increase in ROS concentration with rising LAS levels, with increases of 15.12%, 41.65%, and 59.47% observed at the ROS concentration of LAS-1, LAS-10, and LAS-300, respectively, compared to LAS-0. Across all LAS concentrations, ROS levels in LCM (11.01–17.55 ng/g) were significantly lower than in SCM (14.91–24.35 ng/g) (*p* < 0.01). The results indicate that ROS levels in SCM were highly variable across LAS concentrations, pointing to a weaker antioxidant capacity. However, LCM demonstrated a stable response. Long-term acclimation enhanced LCM’s antioxidant defenses, allowing it to regulate ROS production and accumulation more effectively. Imbalances in ROS concentrations can trigger oxidative stress, increase cytotoxicity, damage cell membrane structure, disrupt nutrient exchange, and ultimately reduce microbial activity [37]. By contrast, the enhanced antioxidant capacity and cellular stability of LCM under high LAS stress underscores the importance of long-term acclimation in promoting microbial resilience to stress.

To further elucidate the impact of LAS on HN-AD microbial metabolism, we measured the ATP content and LDH release rate, as seen in (Figure 2b). ATP, a key energy source for cellular metabolism, showed a direct positive correlation with microbial bioactivity [38]. The ATP content of SCM and LCM decreased with the increase of LAS concentration, particularly at 300 mg/L, where ATP levels dropped to 0.016 μmol/10^6^ cells in SCM and 0.023 μmol/10^6^ cells in LCM. Although ATP levels decrease overall, LCM consistently exhibited higher ATP content than SCM at all LAS concentrations, suggesting a superior energy metabolism capacity. This advantage likely stems may from LCM’s enhanced adaptability, developed long-term acclimation, which enables it to better maintain cell activity and resist the toxic effects of high LAS concentrations. Elevated ATP levels also contributed to restored denitrification performance, highlighting the physiological benefits of long-term acclimation in LCM under stress.

LDH release rate is a marker of cell membrane integrity, as LDH is released into the extracellular matrix when cell membranes are damaged [39]. As LAS concentrations increased from 0 mg/L to 300 mg/L, the LDH release rates of SCM and LCM showed a corresponding increase, reaching 147.87% and 140.97% at LAS-300, respectively. High LAS concentrations correlated with increased cell membrane damage. However, LCM exhibited lower LDH release rates compared to SCM under these conditions, suggesting that LCM maintained better cell membrane integrity and higher cellular activity. These findings suggest that long-term domesticated HN-AD microflora exhibit enhanced antioxidant capacity and cellular activity, contributing to their improved performance under LAS stress. This outcome underscores the crucial role of acclimation in improving physiological resilience, laying the foundation for developing more efficient, robust wastewater treatment technologies under LAS contamination.

### 3.3. Response of EPS to LAS Stress

#### 3.3.1. EPS Content

We also investigated the effects of varying LAS concentrations on the EPS characteristics of microflora with different domestication durations. EPS is a crucial component of activated sludge, playing a vital role in protecting microbial cells from harmful environmental and biotoxic substances [40]. The results indicated distinct EPS secretion by SCM and LCM with changes in LAS concentration. As shown in Figure 2c, LAS at 1 mg/L had minimal impact on EPS secretion in both SCM and LCM compared to LAS-0. However, at 10 mg/L LAS, the EPS content in both SCM and LCM significantly increased to 159.52% and 145.83% of LAS-0 levels (*p* < 0.01), respectively, suggesting that LAS within certain concentration ranges promotes EPS secretion [41]. The slightly higher EPS content in LCM than in SCM suggests that long-term domestication enhances the tolerance of HN-AD microflora to LAS. At higher LAS concentrations (300 mg/L), EPS secretion decreased, with EPS content in SCM and LCM dropping to 64.29% and 85.42% of LAS-0, respectively. This reduction may be attributed to high LAS concentrations altering the structure and chemical properties of EPS, thereby inhibiting its secretion. Despite this, the EPS content in LCM remained higher than in SCM, indicating that long-term domestication contributes to better functional stability under stressful conditions. Polysaccharides (PS) and proteins (PN) are the main components of EPS. These components not only provide a physical barrier for microorganisms but also participate in maintaining the structural stability and functional integrity of cells [42]. The levels of PS and PN followed similar trends as total EPS content with increasing LAS concentrations, with a notable decrease in PN content, particularly at LAS-300. This may be due to changes in osmotic pressure caused by high LAS concentrations, inhibiting the secretion of soluble proteins and compromising the water retention capacity of the cells [43].

To further elucidate the regulatory mechanisms underlying the structural composition of EPS, we calculated the protein-to-polysaccharide (PN/PS) ratio. The results showed that this ratio was consistently higher in the LCM group than in the SCM group, particularly under the LAS-10 condition, reaching 0.428 and 0.396, respectively. This indicates that the long-term acclimated consortium tended to enhance protein secretion to improve stress tolerance. In contrast, the PN/PS ratio in the SCM group dropped significantly to 0.233 under the LAS-300 condition, reflecting a limited ability to modulate EPS composition, which may hinder the maintenance of effective cellular protection under high LAS stress. By comparison, the LCM group maintained relatively high PN/PS ratios across all treatment concentrations (ranging from 0.298 to 0.428), suggesting a more stable and favorable regulation of EPS components. These findings further demonstrate that long-term acclimation not only enhances the overall production of EPS but also optimizes its internal structural composition, serving as a key adaptive strategy for HN-AD microbial consortia in response to surfactant-induced stress.

Our findings also reveal that EPS plays a critical role in the response of HN-AD consortia to LAS stress. Long-term acclimation significantly increased EPS production, enhancing microbial adaptability to environmental stressors. Variations in EPS, PS, and PN, as well as the PN/PS ratio, content indicate an adaptive strategy by which HN-AD consortia maintain cellular stability and function at different LAS concentrations.

#### 3.3.2. Three-Dimensional Fluorescence Characteristics of EPS

We further characterized the composition and structure of EPS using 3D-EEM spectroscopy. The EPS of all treatment groups exhibited strong fluorescence peaks at excitation wavelengths of 275 nm and emission wavelengths of 320–370 nm, indicating significant tryptophan-like components in both SCM and LCM (Figure 2d). Tryptophan-like components, which reflect proteinaceous substances, were identified as part of microbial defense mechanisms. The fluorescence intensity of these tryptophan-like components increased with rising LAS concentrations, suggesting that LAS stress stimulates their secretion by the microflora. Additionally, a moderately strong fluorescence peak was observed in all samples at an excitation wavelength of 340 nm and emission wavelength of 440 nm, indicating the presence of fulvic acid-like substances in the visible region [44]. Fulvic acid-like substances that support EPS structural integrity and microbial aggregation were detected. The presence of humic acid (HA)-like compounds in the metabolites of domesticated HN-AD consortia may enhance microbial tolerance to environmental stress. Notably, at a LAS concentration of 300 mg/L, SCM exhibited higher fulvic acid-like fluorescence compared to other treatment groups, indicating that high concentrations may stimulate the secretion of tryptophan-like and fulvic acid-like components by HN-AD microflora. This observed EPS stimulation likely reflects a response to increased oxidative stress, as these components stabilize cellular structures and provide protection against toxicity. These findings suggest that LAS not only affects the physiological activity of the microflora but also influences the composition and function of EPS by stimulating the secretion of specific metabolites, such as tryptophan-like and fulvic acid-like substances. Long-term domestication of HN-AD microflora under LAS stress may enhance tolerance and environmental adaptability by modulating the chemical composition of EPS. Even at high LAS concentrations, the microflora produced elevated levels of protective metabolites, indicating that long-domesticated HN-AD microflora holds significant potential for treating wastewater containing surfactants and other complex pollutants.

### 3.4. Comparative Heatmap of Stress Markers Under LAS Exposure

To comprehensively evaluate the physiological responses of SCM and LCM microbial consortia under low and high concentrations of LAS stress, seven key stress-related indicators—ROS, ATP, LDH, EPS, PN, PS, and PN/PS ratio—were selected. Z-score normalization was applied to these indicators under two representative treatment conditions (LAS-0 and LAS-300), and an integrated heatmap was constructed for comparative analysis (Figure S1). The results revealed that under LAS-300 stress, the SCM group exhibited typical characteristics of stress-induced damage, including significantly elevated ROS levels, reduced ATP content and EPS production, increased LDH release, and marked decreases in PN and PN/PS ratios. These changes reflect enhanced oxidative stress, impaired energy metabolism, and diminished extracellular structural stability. In contrast, the LCM group maintained relatively low ROS and LDH levels, while exhibiting higher ATP and EPS levels as well as an increased PN/PS ratio, indicating superior oxidative stress buffering capacity and regulation of extracellular protective structures. The trends observed in the heatmap were consistent with the analyses of individual indicators, further emphasizing the critical role of long-term acclimation in strengthening microbial physiological resilience under stress. Collectively, these findings demonstrate that LCM not only maintains advantages in energy metabolism and membrane stability but also exhibits a stronger capacity to regulate both the quantity and composition of EPS. This enables the microbial consortium to better mitigate the multiple toxic effects of high LAS concentrations and maintain overall system stability and denitrification performance.

### 3.5. Analysis of Microbial Community Composition and Diversity

**Figure 3:**
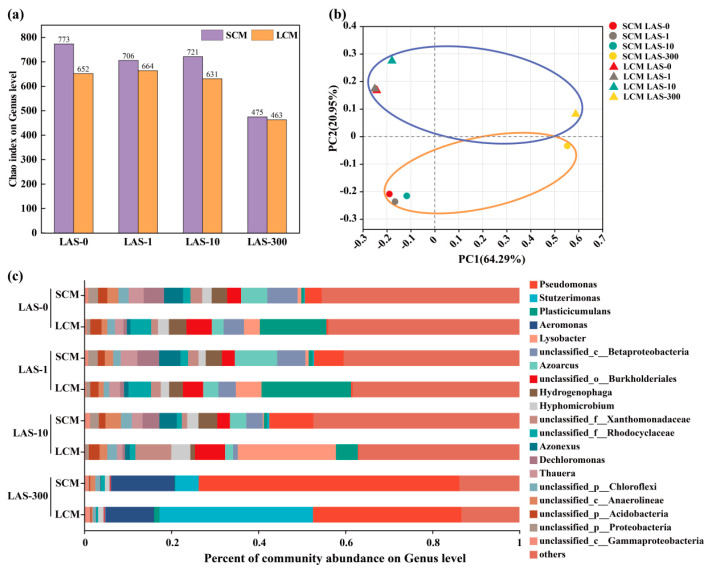
Microbial community composition of SCM and LCM at different LAS concentrations. (**a**) Chao index; (**b**) PCoA (Blue circles: LCM samples; Red circles: SCM samples); (**c**) microbial community composition (top 20 genera).

The community diversity and structural changes in SCM and LCM under different LAS concentrations were analyzed using metagenomic sequencing. The microbial diversity was assessed using the Chao index (Figure 3a), which typically reflects species richness within a community. The Chao index for LCM was consistently lower than that of SCM across all LAS concentrations, indicating that long-term domestication reduced species richness in the sludge microbial community. With increasing LAS concentrations, the Chao index for both SCM and LCM decreased, converging to similar values as many species within the microflora were eliminated. High LAS stress appeared to homogenize the species composition across differently acclimated sludge samples, underscoring its impact on community dynamics. Further analysis of microbial species composition and abundance was conducted using principal coordinate analysis (PCoA), which showed the distribution of bacterial communities at the genus level (Figure 3b). PC1 was 64.29% and PC2 was 20.95%. The results demonstrated distinct differences in the microbial community compositions of SCM and LCM under varying LAS concentrations, with communities occupying different quadrants, indicating a significant effect of domestication time on microbial community structure. However, under 300 mg/L LAS stress, the distance between SCM and LCM communities decreased, reflecting increased similarity in their compositions, consistent with the observed changes in the Chao index. This suggests that high LAS stress levels drive convergence of the microbial communities toward similar structures, regardless of domestication duration.

Analysis of microbial community distribution and proportions at the genus level (Figure 3c) revealed differences in species composition between SCM and LCM at varying domestication durations. In the unstressed environment, *Azoarcus* and unclassified *Betaproteobacteria* were the dominant genera in SCM, with relative abundances of 6.96% and 6.05%, respectively. In contrast, *Plasticicumulans* and unclassified *Burkholderiales* were dominant in LCM, with relative abundances of 15.23% and 5.86%, respectively. At 1 mg/L LAS, the relative abundances of *Azoarcus* and *Plasticicumulans* increased in both SCM and LCM. As LAS concentration further increased, species richness in the microflora declined. At 300 mg/L LAS, *Pseudomonas*, *Aeromonas*, and *Stutzerimonas* were enriched in SCM, with relative abundances of 59.91%, 14.6%, and 5.36%, respectively. These genera were also enriched in LCM but with different relative abundances: 34.12%, 11.14%, and 35.28%, respectively. This indicates that high LAS stress remodels the core species composition within the microbial communities, leading to a rapid convergence of dominant species across different domestication durations.

Previous studies have shown that strains of *Pseudomonas* [45], *Aeromonas* [46], and *Stutzerimonas* [47] not only thrive in extreme environments, such as low temperatures and high salinity, but also show strong environmental adaptability and efficient nitrogen removal capabilities [48,49]. This could explain why, even under 300 mg/L LAS stress, both SCM and LCM maintained high nitrogen removal performance. These findings suggest that under high-stress conditions, genera such as *Pseudomonas*, *Aeromonas*, and *Stutzerimonas* can outcompete others to become dominant members of the microbial community, thereby sustaining the denitrification function and overall stability of the system. Moreover, certain genera demonstrated a capacity to degrade LAS, which may explain their dominance under high LAS conditions. Their adaptability and stability in high-stress environments suggest that long-term domesticated microbial communities hold promise for treating highly concentrated LAS wastewater.

### 3.6. Nitrogen Metabolism

#### 3.6.1. Changes in the Abundance of Nitrogen Cycling Functional Genes

**Figure 4:**
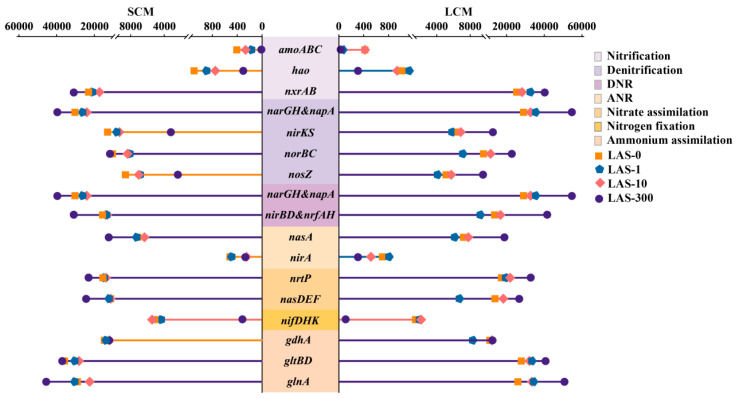
Abundance of nitrogen functional genes in SCM and LCM at different LAS concentrations.

By the end of the experiment, numerous genes related to nitrogen cycling were detected in both SCM and LCM samples exposed to different LAS concentrations, as seen in (Figure 4). These genes are involved in several key stages of the nitrogen cycle, including nitrification, denitrification, dissimilatory nitrate reduction (DNR), assimilatory nitrate reduction (ANR), nitrogen fixation, and ammonia assimilation. During nitrification, the *amoABC* and *hao* genes encode for ammonia monooxygenase (*amo*) and hydroxylamine dehydrogenase (*hao*), respectively, which are essential enzymes for converting NH_4_^+^ to NO_2_^−^ [6]. The results showed that the abundances of *amoABC* and *hao* genes were reduced in both SCM and LCM as LAS concentrations increased from 0 mg/L to 300 mg/L, indicating that high LAS stress inhibits the activity of these enzymes, thereby limiting NH_4_^+^ consumption. This suggests that the nitrification pathway is particularly susceptible to LAS inhibition. Enzymatic activity crucial for nitrification was significantly impaired as LAS concentration increased. The reduced abundance of nitrification-related genes suggests that LAS not only hinders enzyme functionality but may also affects nitrifying bacterial viability, likely through oxidative stress and membrane damage. *Amo* and *hao* genes, essential for ammonia-to-nitrite conversion, showed reduced activity, thereby impairing the first step of nitrification. This inhibition may lead to ammonia accumulation, which is toxic to many microorganisms and compromises overall nitrogen removal efficiency. The observed differences between SCM and LCM suggest that long-term domestication confers some resilience to LAS inhibition, although not enough to fully counteract high-LAS toxicity. In the denitrification pathway, the absolute abundances of *nxrAB* (NO_2_^−^ → NO_3_^−^), *narGH*, *napA* (NO_3_^−^ → NO_2_^−^), and *norAB* (NO → N_2_O) genes increased under high LAS concentrations, and the gene abundance in LCM was generally higher than that in SCM. At 300 mg/L LAS, the abundances of *nirKS* (NO_2_^−^ → NO) and *nosZ* (N_2_O → N_2_) were also higher in LCM compared to SCM, suggesting that long-term domestication enhances denitrification activity under high LAS stress. The denitrification process may play an essential role in mitigating LAS toxicity, especially in domesticated microflora. Regarding NH_4_^+^ production, gene abundance levels associated with DNR and ANR processes were elevated, explaining the higher NH_4_^+^-N concentrations observed in the later stages of the denitrification assay. Notably, the activities of enzymes encoded by *nirBD* and *nrfAH* were much higher than those encoded by *nirA* in both SCM and LCM, highlighting the dominance of the DNR pathway in nitrate reduction. The ATP-dependent transporter protein encoded by *nasDEF* and the proton motive force-dependent transporter protein encoded by *ntrP* are crucial for synergistic nitrate assimilation [50]. This suggests that dissimilation reduction plays a critical role in nitrogen balance under LAS stress. The abundance of *nasDEF* and *ntrP* genes in SCM and LCM remained unaffected by increasing LAS concentrations, indicating that elevated LAS levels did not negatively impact nitrate transmembrane transport. NH_4_^+^-N can be reintroduced into the nitrogen metabolism during the HN-AD process or assimilated via pathways involving glutamine synthetase, glutamate synthetase, and glutamate dehydrogenase [51]. Numerous genes related to ammonia assimilation, such as *gdhA*, *gltBD,* and *glnA*, were detected in both SCM and LCM, with their abundance showing small changes in response to varying LAS concentrations. This suggests that the ammonia assimilation process remains active even under high LAS stress conditions. Additionally, nitrogen-fixing enzymes (*nif*) were present in both SCM and LCM under lower LAS concentrations; however, their activity declined with increasing LAS levels, indicating that high LAS concentrations adversely affect nitrogen fixation.

#### 3.6.2. Regulation of Nitrogen Metabolism and Core Species

**Figure 5:**
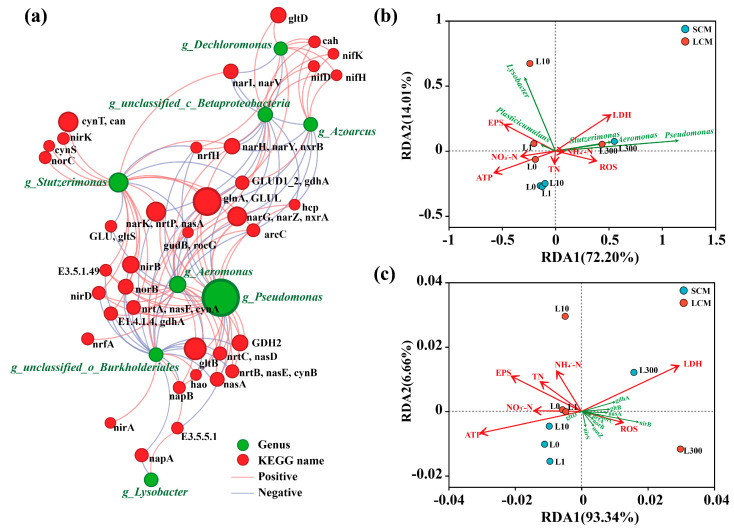
(**a**) Network correlation analysis of nitrogen-metabolizing species and functional genes (Pearson’s correlation coefficient > 0.5, *p* < 0.05). Redundancy analyses based on the relationship between (**b**) key genera; (**c**) nitrogen functional genes and environmental variables in biological samples. L0, L1, L10, and L300 denote LAS-0, LAS-1, LAS-10, and LAS-300, respectively.

To explore the relationship between nitrogen-cycling species and nitrogen-functional genes and to clarify the distribution of nitrogen-cycling genes, a network correlation map was constructed between the top 10 genera by total abundance and the top 50 nitrogen-functional genes (Figure 5a). The results showed that the most abundant genera, *Pseudomonas*, *Stutzerimonas*, and *Aeromonas*, were significantly positively correlated with 18, 16, and 19 functional genes, respectively. These genes include many related to nitrification, denitrification, dissimilatory nitrate reduction, and ammonia assimilation, suggesting that these genera are key contributors to nitrogen cycling.

Further analysis of the contributions of different species to essential nitrogen metabolism genes revealed that the structure of core species and functional genes was remodeled with the change of domestication time (Figure S2). In the bacterial communities of SCM and LCM, *Nitrospira* was the primary host encoding *amo* at LAS concentrations of 0–1 mg/L, while *Hyphomicrobium*, responsible for encoding *hao*, was present in small amounts across all LAS environments. The core host of nitrite oxidoreductase (*nxr*), a key enzyme in nitrification, varied between SCM and LCM across different LAS concentrations. At LAS concentrations of 1–10 mg/L, unclassified *Burkholderiales* was the core host in both SCM and LCM, and *Plasticicumulans* was the core host in LCM. However, at 300 mg/L LAS, the abundance of unclassified *Burkholderiales* and *Plasticicumulans* decreased significantly. *Pseudomonas* became the major contributor to *nxr* with high abundance. The core hosts of membrane-bound nitrate reductase (*nar*), periplasmic nitrate reductase (*nap*), and assimilatory nitrate reductase (*nas*), which catalyze the conversion of NO_3_^−^ to NO_2_^−^, also varied with LAS concentration. At LAS concentrations of 0–10 mg/L, the key genes encoding these three enzymes differ in the core host species of SCM and LCM. The core host species of *narG* in both SCM and LCM was unclassified *Burkholderiales*. The core hosts of *napA* in SCM and LCM were *Azonexus* and unclassified *Rhodocyclaceae*, respectively. The core hosts of *nasA* in SCM were *Azonexus* and *Hydrogenophaga*, while the core hosts in LCM are *Plasticicumulans* and *Chloracidobacterium*. At 300 mg/L LAS, *Pseudomonas* became the dominant contributor to *narG*, *napA*, and *nasA*, similar to its role with *Nxr*. Other key genes involved in the denitrification process, such as *nirK*/*S*, *norB*/*C*, and *nosZ*, were predominantly hosted by *Pseudomonas*, *Stutzerimonas*, *Dechloromonas*, unclassified *Betaproteobacteria*, unclassified *Chloroflexi*, *Azonexus*, and *Azoarcus* in SCM and by *Pseudomonas*, *Stutzerimonas*, unclassified *Burkholderiales*, unclassified *Rhodocyclaceae*, unclassified *Chloroflexi*, unclassified *Thermoleophilia*, and *Plasticicumulans* in LCM. Notably, *Pseudomonas* and *Stutzerimonas* had higher contributions to these genes in high LAS environments. The core host species of *nirB*/*D*, encoding key enzymes in the reduction of NO_2_^−^ to NH_4_^+^, differed between SCM and LCM. In SCM, *Pseudomonas*, *Aeromonas*, *Stutzerimonas*, *Azoarcus*, and *Dechloromonas* were core hosts, while in LCM, *Pseudomonas*, *Aeromonas*, *Stutzerimonas*, *Plasticicumulans*, and unclassified *Rhodocyclaceae* were predominant. The abundance of *Pseudomonas*, *Aeromonas*, and *Stutzerimonas* significantly increased at 300 mg/L LAS compared to lower concentrations. The major contributor to encoding *nirA* was unclassified *Acidobacteria*, which was only minimally present in the treatment group with LAS concentrations of 0–10 mg/L for both SCM and LCM. The key genes for ammonia assimilation, *gltB*/*D* and *glnA*, remained abundant across different treatment groups in both SCM and LCM, though the core host composition varied. At low LAS concentrations (0–10 mg/L), *Plasticicumulans*, unclassified *Betaproteobacteria*, *Dechloromonas*, and *Lysobacter* were dominant hosts. However, at 300 mg/L LAS, *Pseudomonas*, *Stutzerimonas*, and *Aeromonas* became the primary contributors with significantly higher abundances. The gene *nifD*/*K*/*H*, encoding nitrogen-fixing enzymes, was mainly associated with *Azoarcus*, *Dechloromonas*, and *Azonexus* and was present in low abundance in the LAS-0, LAS-1, and LAS-10 treatment groups of both SCM and LCM.

Overall, these results indicate that LAS stress induces significant shifts in the composition and function of core species involved in nitrogen cycling within the HN-AD microflora. In particular, the genera *Pseudomonas*, *Aeromonas*, and *Stutzerimonas* emerged as dominant contributors in key nitrogen cycle steps, highlighting their adaptability and tolerance to high LAS environments. These microbes effectively perform processes such as denitrification and ammonia assimilation even at high LAS concentrations, ensuring sustained nitrogen removal and reducing nitrogen-containing gas emissions. Their increased resistance to oxidative stress and cell damage enables them to maintain cellular activity and functional stability, thus preserving the overall nitrogen removal efficiency of the system under high pollution loads.

### 3.7. Correlations Between Environmental Variables, Bacterial Communities, and Nitrogen Functional Genes

Redundancy analysis (RDA) was conducted to evaluate the relationships between bacterial communities and environmental variables at different LAS concentrations (Figure 5b). The results showed that *Plasticicumulans* and *Lysobacter* were positively correlated with EPS content in LCM under low LAS stress (0–10 mg/L) (*p* < 0.01). This indicates that long-term domesticated HN-AD microflora adapt to low-toxicity environments by increasing EPS content when exposed to low LAS concentrations. Results further suggest that at low LAS concentrations, EPS secretion acts as an essential adaptive mechanism for microbial resistance by forming a physical barrier to protect against toxic external substances. At a LAS concentration of 300 mg/L, the dominant bacteria in both SCM and LCM (*Pseudomonas*, *Stutzerimonas*, and *Aeromonas*) showed significant positive correlations with LDH, ROS, and NH_4_^+^-N concentrations (*p* < 0.01). High LAS concentrations, however, increase cell membrane damage and oxidative stress, impairing ammonia nitrogen removal rates.

This indicates that while high concentrations promote the enrichment of these genera and reduce differences in community structure between SCM and LCM, they also impair the intracellular anti-oxidative stress response, cause cellular damage, and reduce denitrification performance.

We also assessed the correlations between key nitrogen functional genes and environmental variables using RDA analysis (Figure 5c). The analysis revealed that many nitrogen functional genes (e.g., *nasA*, *norBC*, *nosZ*, *nirB*, *gltB*) were significantly negatively correlated with ATP, EPS, and nitrogen-related metrics (TN, NH_4_^+^-N, and NO_3_^−^-N), while showing positive correlations with LDH and ROS *(p* < 0.01). These genes were predominantly concentrated in the LCM at LAS-300.

Nitrifying and denitrifying microorganisms are known for their ability to biotransform environmental micropollutants [52,53,54]. Thus, while high LAS concentrations degrade the survival environment for HN-AD consortia, long-term acclimation improves LCM’s functional resilience. Decreases in ATP and EPS content signal LAS-induced cellular damage, yet nitrogen removal rates increase, suggesting that high LAS may enhance LCM’s environmental adaptability by selecting and enriching functional genes related to oxidative stress tolerance and efficient nitrogen metabolism. Consequently, LCM can effectively sustain both biodegradation and nitrogen removal functions under LAS stress. Overall, the domestication time significantly influenced the composition, functional properties, and interactions of microbial communities with environmental variables. These findings offer important mechanistic insights into how domestication time impacts the functional properties and environmental adaptations of microbial communities. They also serve as a reference for optimizing microbial communities under various environmental stresses.

## 4. Conclusions

This study systematically evaluated the denitrification performance of HN-AD microbial consortia under LAS stress and explored their community evolution and functional response mechanisms under different acclimation periods. Compared with short-term acclimation, long-term acclimation (LCM) significantly enhanced microbial tolerance to LAS, resulting in more stable denitrification efficiency and physiological adaptability. Under 300 mg/L LAS, the TN removal efficiency of LCM reached 97.40%, which was 16.89% higher than that of SCM. At the physiological level, long-term acclimation alleviated oxidative stress by enhancing ROS scavenging, increasing ATP production, maintaining cell membrane integrity, and promoting extracellular polymeric substances (EPS) secretion. Metagenomic analysis revealed that LCM selectively enriched key tolerant genera such as *Pseudomonas*, *Aeromonas*, and *Stutzerimonas* under LAS stress, and maintained higher expression levels of functional genes involved in denitrification and ammonium assimilation, highlighting their dominant roles in nitrogen cycling. Although high LAS concentrations reduced the diversity gap between SCM and LCM communities, LCM still exhibited greater functional resilience and recovery capacity. Network and redundancy analyses further revealed the coupling relationships among microbial communities, functional genes, and environmental factors, emphasizing the role of long-term acclimation in reshaping microbial stress response mechanisms. It should be noted that all experiments in this study were conducted under well-controlled laboratory conditions, with temperature, pH, and dissolved oxygen levels maintained consistently. Therefore, although our findings demonstrate the positive effects of acclimation on microbial adaptation, whether similar stability and functionality can be sustained in full-scale wastewater treatment systems under complex water quality conditions remains to be validated through further field investigations and engineering evaluations. Overall, this study provides a theoretical foundation for the optimization of microbial consortia and technical support for biological nitrogen removal in wastewater containing high concentrations of surfactants.

## Data Availability

The original contributions presented in the study are included in the article/Supplementary Material, further inquiries can be directed to the corresponding author.

## Supplementary Materials

The following supporting information can be downloaded at: https://www.mdpi.com/article/10.3390/microorganisms13051031/s1, Figure S1: Comparative Heatmap of Stress Markers under LAS Exposure; Figure S2: Nitrogen metabolic pathways and core hosts.
