# Supplementary material for: Acclimation Time Enhances Adaptation of Heterotrophic Nitrifying-Aerobic Denitrifying Microflora to Linear Anionic Surfactant Stress
Source: Microorganisms. 2025 Apr 29;13(5):1031. doi: 10.3390/microorganisms13051031 (PMC12114585; doi:10.3390/microorganisms13051031)
Supplement: Supplementary file 1 [file microorganisms-13-01031-s001.zip › microorganisms-3565700-supplementary.pdf]

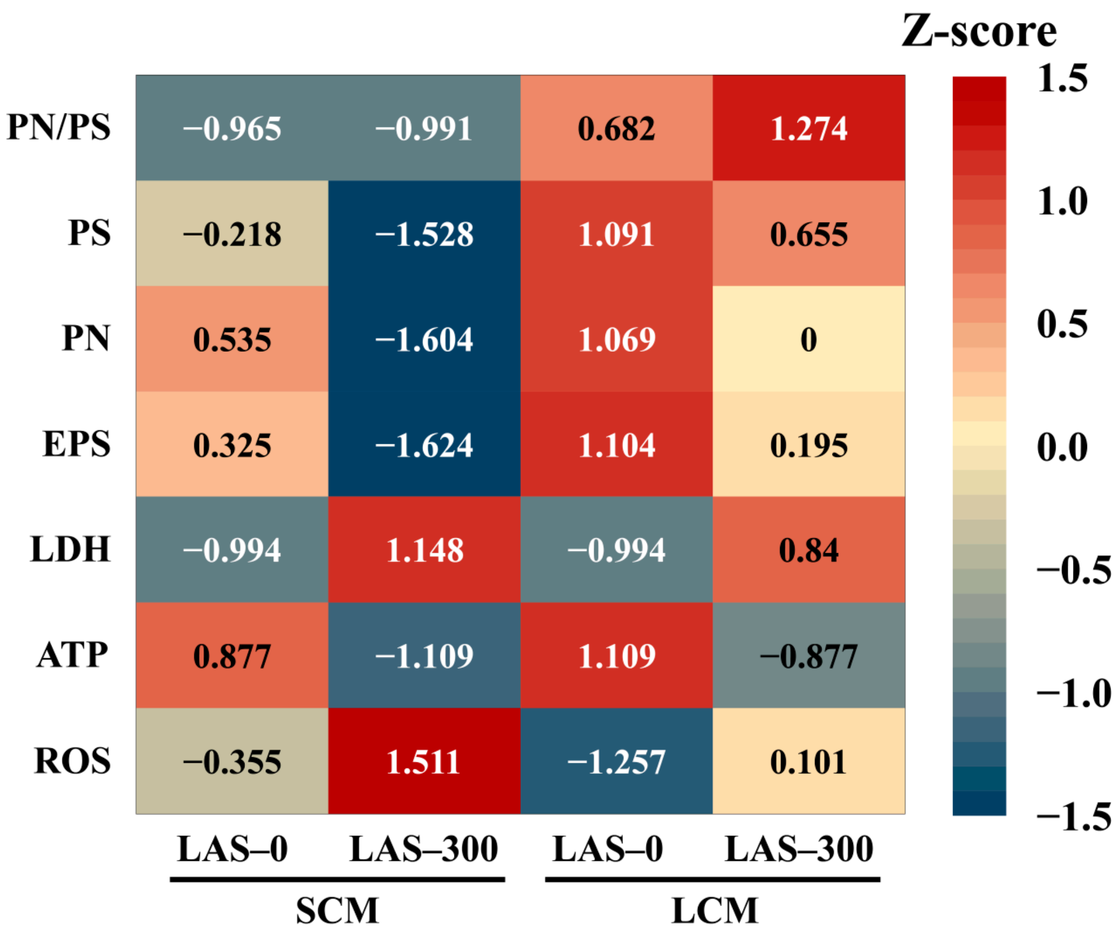

Fig. S1 Comparative Heatmap of Stress Markers under LAS Exposure

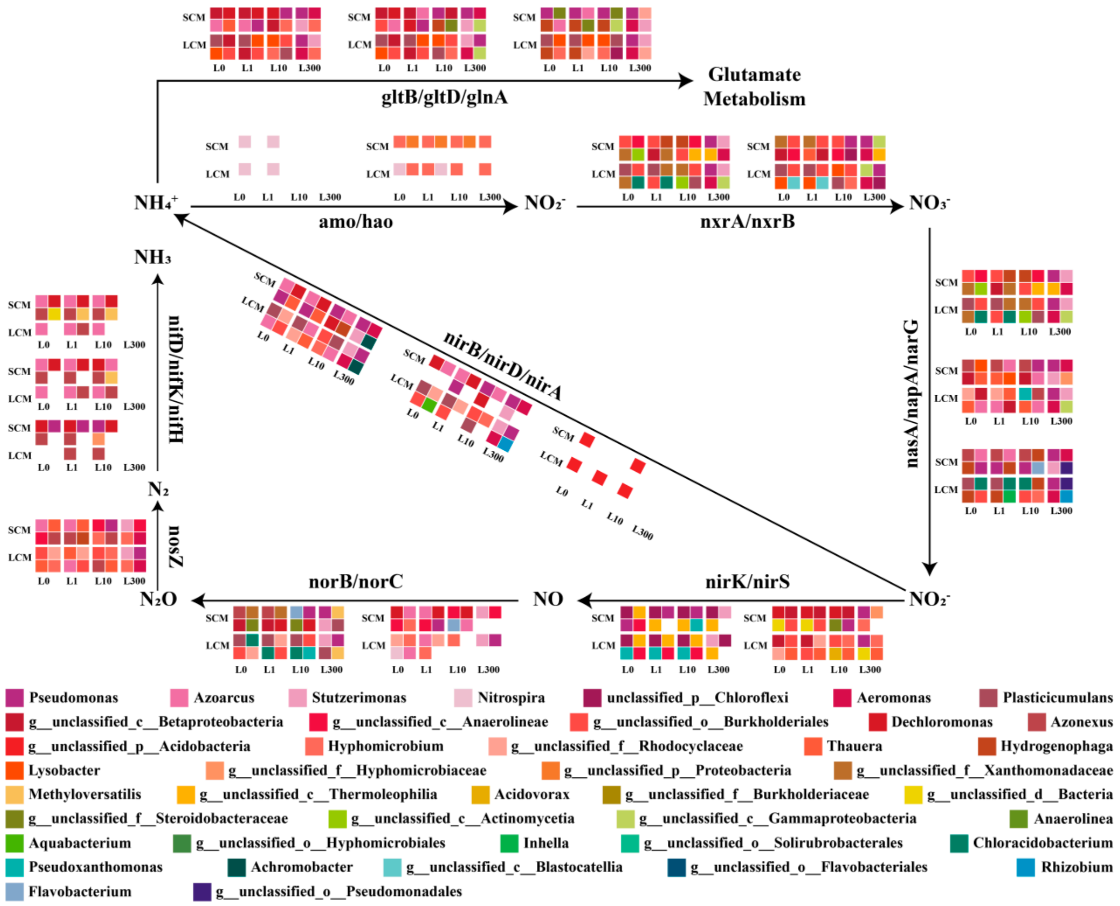

Fig. S2 Nitrogen metabolic pathways and core hosts
